# Effects of the SmartACT Intervention on Motor and Psychological Variables in Adolescent Athletes: A Controlled Trial Using BlazePod and Microgate

**DOI:** 10.3390/children12101338

**Published:** 2025-10-05

**Authors:** Barabási Madár Timea, Costea-Bărluţiu Carmen, Ordean Mircea Nicolae, Mancini Nicola, Grosu Vlad Teodor, Sabău Anca Maria, Popovici Cornelia, Carlos Hervás-Gómez, Grosu Emilia Florina, Monea Dan

**Affiliations:** 1 Faculty of Physical Education and Sport, “Babes-Bolyai” University, Mihail Kogălniceanu 1, 400347 Cluj-Napoca, Romania; timea.barabasi@ubbcluj.ro (B.M.T.); emilia.grosu@ubbcluj.ro (G.E.F.); dan.monea@ubbcluj.ro (M.D.); 2 Faculty of Psychology and Educational Sciences, “Babes-Bolyai” University, Sindicatelor 7, 400347 Cluj-Napoca, Romania; carmen.costea@ubbcluj.ro; 3 Department of Physical Education and Sports, “December 1, 1918” University, Gabriel Bethlen 5, 510009 Alba Iulia, Romania; 4 Department of Science of Education and Sport, Pegaso Telematic University, 80143 Naples, Italy; 5 Faculty of Industrial Engineering, Robotics, and Production Management, Technical University of Cluj-Napoca, Muncii 103-105, 400461 Cluj-Napoca, Romania; vlad.grosu@mdm.utcluj.ro; 6 Departament of Physical Education and Sport and Kinetotherapy, Faculty of Geography, Turism and Sport, University of Oradea, 410087 Oradea, Romania; sabauancamaria@yahoo.com; 7 Physical Education Discipline, University of Medicine and Pharmacy “Iuliu Hațieganu”, Pasteur 6, 400347 Cluj-Napoca, Romania or cornelia.popovici@umfcluj.ro; 8 Department of Teaching and Educational Organization, University of Sevilla, 41013 Sevilla, Spain; hervas@us.es

**Keywords:** SmartACT, adolescent athletes, agility, reaction time, DASS-21, GMSCS, ACT, hypnosis, performance psychology

## Abstract

**Background/Objectives**: Agility and reaction speed are critical components of sports performance and are influenced by both physical conditioning and psychological state. Interventions such as SmartACT, which integrate mindfulness, acceptance, and commitment, guided imagery and hypnosis techniques are still underexplored in high-performance sport, despite their potential to affect both psychological and motor dimensions. **Methods**: This 7-week controlled trial investigated the effectiveness of SmartACT in reducing psychological and somatic symptoms and enhancing motor performance in adolescent athletes. A total of 193 athletes aged 15–18 years were assigned to three groups: SmartACT (*n* = 69), MAC (*Mindfulness–Acceptance–Commitment*, the standardized Gardner & Moore protocol; *n* = 65), and a control group (*n* = 59). Agility was measured using the T-Drill Agility Test with Microgate electronic timing, and reaction speed was assessed using BlazePod devices. Psychological and somatic symptoms were evaluated using the Depression, Anxiety, and Stress Scale (DASS-21) and the Ghent Multidimensional Somatic Complaints Scale (GMSCS). **Results**: The SmartACT group showed significantly improved agility (*MD* = −1.07 s, *p* < 0.001, *d* = 2.50, 95% CI [1.79, 3.35]), faster reaction times (*MD* = −643.75 ms, *p* < 0.001, *d* = 0.85, 95% CI [0.35, 1.41]), and a higher number of BlazePod touches (*MD* = +2.53, *p* < 0.001, *d* = 1.43, 95% CI [0.87, 2.07]). Psychological symptoms (DASS-21) and somatic complaints (GMSCS) decreased significantly more than in the MAC and control groups. **Conclusions**: SmartACT appears to be an effective hybrid psychological intervention to simultaneously improve physical performance and reduce psychological and psychosomatic distress in adolescent athletes.

## 1. Introduction

Early participation in organized sport contributes to psychophysical development and long-term well-being in youth [1,2]. Agility is a complex skill that integrates coordination, speed, and rapid decision-making with perceptual–cognitive processes such as visual scanning and anticipation; it is a key determinant of performance in open-skill team sports where athletes must respond to unpredictable stimuli [3,4,5,6,7,8,9,10,11,12,13]. Contemporary accounts emphasize reactive agility—changes in direction in response to sport-specific cues—because it better captures real game demands and predicts talent more accurately than pre-planned agility [5,7,14], with implications for identification and development pathways [4,6,7,15].

Across ball and racket sports, rapid reactions and efficient visual processing underpin success under tactical pressure [4,15,16,17]. Accordingly, reaction time (RT) is not only a marker of performance potential but is tightly coupled with reactive-agility outcomes and decision speed in ecologically valid tasks [4,5,6,7,15]. Considering this linkage, the present study treats RT as a core motor outcome alongside agility, as both reflect the integration of perceptual, cognitive, and motor processes that our intervention seeks to influence.

Beyond motor performance, adolescent sport participation is associated with psychological benefits—including improved self-esteem and lower symptoms of depression and anxiety—whereas stress and anxiety can contribute to somatic complaints (e.g., fatigue, pain, gastrointestinal discomfort) that interfere with training and competition [18,19,20,21,22,23,24,25,26,27,28,29,30,31,32,33,34,35,36,37,38,39]. In applied settings where maximal testing is not always feasible, combining quick motor tests with validated questionnaires such as the Ghent Multidimensional Somatic Complaints Scale (GMSCS) provides a practical screen for psychophysiological imbalance [40,41].

Interest in psychological methods that target attention, emotion regulation, and imagery has grown in performance sport. Mindfulness- and acceptance-based approaches, guided imagery, and hypnosis have been linked to reduced psychosomatic symptoms and improved execution under pressure [42,43,44,45,46,47,48,49,50,51,52,53,54,55,56,57,58,59,60,61,62]. However, the SmartACT protocol—integrating mindfulness, acceptance, commitment, guided imagery, and hypnosis—has not been systematically tested in adolescent athletes on both motor and psychological outcomes simultaneously, leaving a relevant gap in the literature.

### Aims and Hypotheses

This study aims to evaluate the effects of a 7-week SmartACT intervention on both motor performance (agility, reaction time) and psychological well-being (anxiety, depression, somatic symptoms) in adolescent athletes. Based on prior literature, we hypothesize that SmartACT will produce greater improvements than the MAC and control conditions across all measured outcomes.

## 2. Materials and Methods

This study followed a quasi-experimental, controlled, repeated-measures design with pretest and posttest assessments over seven weeks. A total of 193 student athletes (age 15–18 years, M = 16.34; SD = 0.64) were recruited from sports-focused high schools in Transylvania (Romania) and educational centers in Hungary (Table 1). Participants were assigned to the three groups (SmartACT, MAC, Control) using cluster allocation at the school or training-group level, rather than individual randomization, which justifies the quasi-experimental design. Inclusion criteria were: (i) age between 15 and 18 years, (ii) participation in structured sports training at least twice per week, and (iii) informed consent from both athletes and their guardians. Exclusion criteria included: (i) current musculoskeletal or neurological injuries, (ii) ongoing psychological treatment, or (iii) incomplete participation in pre- or post-assessments.

**Table 1:** Participant characteristics at baseline (N = 193).

Characteristic	Category	*n* (%)
Age (years), mean ± SD	—	16.34 ± 0.64
Primary sport	Volleyball 46 (23.9%); Basketball 45 (23.4%); Football 42 (21.8%); Handball 20 (10.2%); Athletics 13 (6.6%); Wrestling 12 (6.1%); Others ≤ 2% each	
Sports experience	>4 years 107 (55.3%); 3–4 years 26 (13.7%); 1–2 years 44 (22.8%); 1st year 16 (8.1%)	
Training frequency	≥5×/week 105 (54.3%); 4×/week 43 (22.3%); 2–3×/week 43 (22.3%); 1×/week 2 (1.0%)	
Additional sports	Yes 66 (34.0%); No 127 (66.0%)	
Diet pattern	Balanced 89 (46.0%); High-protein 58 (29.9%); Carbohydrate-dominant 28 (14.7%); Sugar-rich 6 (3.3%); Not reported 12 (6.2%)	

Note. *n* = number of participants; % = percentage; *SD* = standard deviation.

Participants completed an assessment that addressed both motor and psychological components. From a motor perspective, agility was tested using the T-Drill agility task using the Microgate electronic timing system, and reaction speed was assessed using BlazePod devices, which involve rapid responses to visual stimuli. Psychologically, participants completed two validated instruments: the Depression, Anxiety, and Stress Scale (DASS-21) [63], which measures depression, anxiety, and distress, and the Ghent Multidimensional Somatic Complaints Scale (GMSCS) [64], which assesses the presence and intensity of psychosomatic symptoms.

### 2.1. Motor Assessment: Agility and Reaction Speed

Before testing, all participants completed a 10 min standardized warm-up under supervision, including dynamic stretches, joint rotations, and functional drills designed to activate major muscle groups [65]. The T-Drill agility protocol was demonstrated and explained to ensure consistent execution.

**Figure 1:**
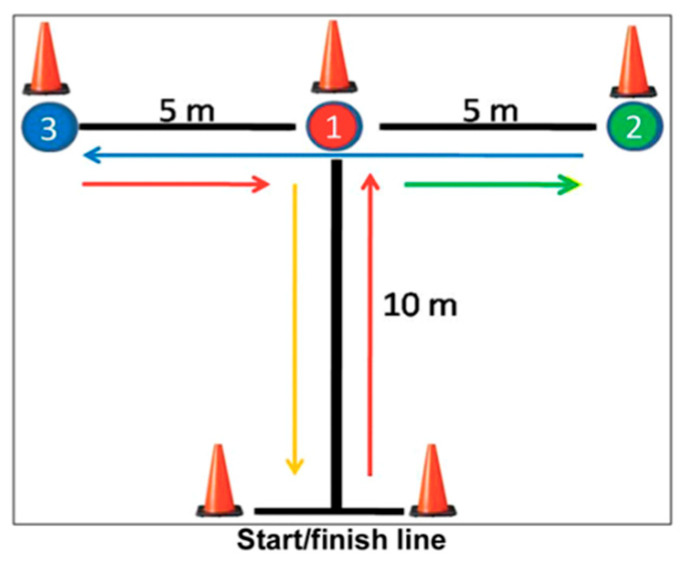
T-Drill Agility Test.

**Figure 2:**
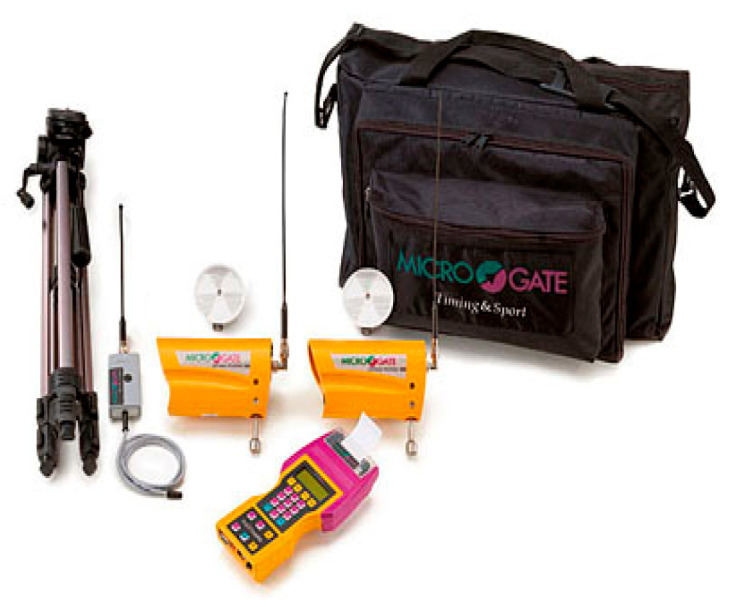
Microgate equipment kit Racetime Light Radio.

A total of 168 participants performed the T-Drill Agility Test (Figure 1), applied both in the pre-test and post-test stages. This test assesses movement speed, the ability to quickly change direction and motor coordination, in conditions that require quick reactions and precision. The T-Drill agility has been shown to have high test–retest reliability and validity in athletic populations [66]. The execution time was recorded using a Microgate Racetime Light Radio electronic timing system (Microgate, Bolzano, Italy; Figure 2), installed at the start–finish line to ensure the objectivity of the measurements. The route was visually delimited by colored cones, which indicated the directions of movement and the mandatory touch points, according to the sequence provided by the standard T-Drill agility protocol.

**Figure 3:**
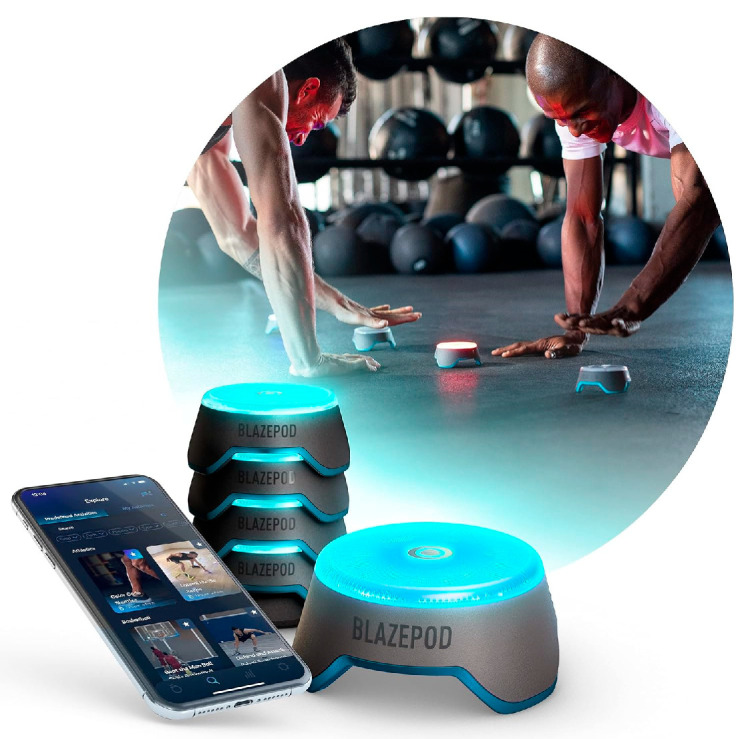
BlazePod Ltd. (www.blazepod.eu).

A total of 185 participants were assessed in terms of reaction speed and total number of touches using the BlazePod training and testing system (BlazePod Ltd., Tel Aviv, Israel; Figure 3), both in the pretest and posttest stages. BlazePod is a modern motor assessment and training system (BlazePod Ltd., Tel Aviv, Israel), designed to develop and measure psychomotor functions through visual light stimuli, emitted by portable LED modules. In this study, a set of 12 modules was used, controlled via the BlazePod mobile application (www.blazepod.eu, accessed on 5 April 2025), installed on a phone compatible with the iOS operating system. The reliability and validity of this system in assessing reactive agility have already been confirmed in the specialized literature, being frequently used in sports testing contexts [67,68]. The devices were configured to allow simultaneous testing of six participants. Each athlete was assigned a personal code entered in the application, allowing individual monitoring of performance. The motor exercise used, called “Color Catch”, involved the rapid reaction of the participants by touching visually activated color modules. The 12 pods were placed on adjustable tripods at a height of 90–120 cm, arranged in a semicircle of ~3 m in front of the participant to allow multidirectional reaching. Pods were spaced 50–70 cm apart to require lateral and forward movements. The testing was carried out in two consecutive rounds of 60 s each, separated by a 30 s break. Instructions were clearly communicated before the start of the test, and the activity was standardized. Performance was monitored in real time by the application, which automatically recorded parameters such as the total number of touches and the average reaction speed.

### 2.2. Psychological and Psychosomatic Assessment

A total of 193 participants completed the Depression, Anxiety, and Stress Scales (DASS-21) [63] and the Ghent Multidimensional Somatic Complaints Scale (GMSCS) [64] at both pre- and post-test assessments. The Depression, Anxiety and Stress Scale (DASS-21) is a quantitative instrument used to assess depression, anxiety and stress. Based on the scores obtained, individuals can be classified into five severity categories for each subscale: normal, mild, moderate, severe and extremely severe.

The questionnaire consists of 21 items, and respondents rate the frequency with which they have experienced certain states in the past week, using a scale from 0 (“not at all”) to 3 (“very much or most of the time”) [63].

The Ghent Multidimensional Somatic Complaints Scale (GMSCS) was used to assess symptoms in experimental subjects. They rated 18 somatic symptoms that had occurred in the past four weeks, in terms of frequency and intensity, using a scale from 0 to 7. Frequency was rated as follows: 0 = never, 7 = constantly; and intensity: 0 = not at all, 7 = unbearable.

The scale includes five subscales: pain localized to the head, shoulders and stomach, sensations of cold or heat, cardiac manifestations and fatigue [64]. Both scales were linguistically adapted in Romanian [69,70,71] and Hungarian [69,70,71] languages.

### 2.3. Participant Flow and Outcome Completion

Of the 193 athletes initially enrolled, completion rates differed across outcome measures due to occasional absences or missing data. A total of 168 completed the T-Drill agility, 185 completed the BlazePod tests, and all 193 completed the DASS-21 and GMSCS questionnaires (Table 2). No adverse events or intervention-related withdrawals were reported.

**Table 2:** Participant flow by outcome (pre–post analyzed).

Outcome	Baseline Enrolled (N)	Completed Pre–Post (n)	Missing (n)	Reason for Missing
T-Drill agility	193	168	25	Absence or incomplete timing data
BlazePod tests	193	185	8	Device availability/missed session
DASS-21	193	193	0	—
GMSCS	193	193	0	—

Note. Reasons for non-completion were administrative or logistical (e.g., absence, missing data, device unavailability). No adverse events or intervention-related withdrawals were reported. *N* = total number of participants enrolled at baseline; *n* = number of participants; — = not applicable.

### 2.4. Blinding

Outcome assessors were not blinded to group assignment. However, motor outcomes were recorded using automated and objective systems (Microgate electronic timing, BlazePod application), and standardized procedures were applied across groups to minimize detection bias.

The study was built on a repeated measures design, including an initial assessment (pretest) and a subsequent assessment (posttest), conducted at an interval of seven weeks. Participants were divided into three experimental groups. The first group, SmartACT (*n* = 69, 35.8%), benefited from a structured psychological program, carried out over seven weeks, which integrated principles of acceptance and commitment therapy (ACT) with hypnosis and guided imagery techniques, adapted to the specifics of sports activity. The second group, MAC (Mindfulness–Acceptance–Commitment), consisting of 65 participants (33.7%), followed the standardized protocol proposed by Gardner and Moore (2007) [72], focused on developing full awareness of internal experiences, their acceptance and orientation towards action in accordance with one’s own values. The control group (*n* = 59, 30.6%) continued their usual sports and educational activity, without participating in any specific psychological intervention during the study.

It is assumed that there are significant relationships between motor performance and psychological indicators, such that a more balanced level of emotional state and a more functional body perception correlate with superior motor efficiency. In this context, it is considered that the general psychological state and somatic perceptions of adolescent athletes significantly influence motor performance and psychomotor reactivity. It is also anticipated that the SmartACT psychological intervention, which combines mindfulness, acceptance and commitment techniques, guided imagery and hypnotherapy, will lead to significant improvements in both physical (agility, reaction speed) and psychological aspects, by reducing symptoms of depression, anxiety, stress and psychosomatic symptoms. The SmartACT group is expected to obtain significantly higher scores in the posttest stage, compared to the MAC group and the control group, which would support the effectiveness of a hybrid intervention in simultaneously optimizing psychological functioning and motor performance in a sports context.

### 2.5. Statistical Analysis

Data were analyzed using SPSS Statistics v25 (IBM, Armonk, NY, USA). Descriptive statistics are reported as mean ± SD (with 95% confidence intervals where relevant). Pearson’s *r* quantified bivariate associations. Within-group changes were tested with paired-samples *t*-tests; time × group effects were examined with a mixed repeated-measures ANOVA (within-subject factor: time [pre, post]; between-subject factor: group [SmartACT, MAC, Control]). When appropriate, between-group pairwise comparisons at posttest used Tukey–Kramer HSD. All tests were two-tailed with α = 0.05.

Effect sizes were calculated as Cohen’s *d* for *t*-tests and partial eta squared (η^2^p) for ANOVAs, each with 95% confidence intervals. Benchmarks were: Cohen’s *d* small = 0.20, medium = 0.50, large = 0.80; Pearson’s *r* small = 0.10, medium = 0.30, large = 0.50; η^2^p small = 0.01, medium = 0.06, large = 0.14. Assumptions of normality and homogeneity of variances were checked with Shapiro–Wilk (and Q–Q plots) and Levene’s tests; with two time points, sphericity is not applicable. Analyses were performed per outcome using all available paired observations (pairwise deletion).

## 3. Results

### 3.1. Analysis of Correlations Between Motor Variables, Perception of Somatic Symptoms and Indicators of Psychological Distress

Table 3 reports significant correlations between motor performance (T-Drill agility, BlazePod Hits, and Reaction Time) and scores from the DASS-21 and GMSCS, which assess psychological distress and psychosomatic symptoms.

T-Drill agility times were negatively correlated with BlazePod Hits (*r* = −0.400, *p* < 0.01), indicating a moderate-to-strong association whereby faster agility was linked to more effective visual–motor responses. BlazePod Hits and Reaction Time were strongly and inversely related (*r* = −0.894, *p* < 0.01), confirming that increased reactivity corresponds to shorter response times. T-Drill agility times were also positively associated with several GMSCS subscales, especially fatigue (*r* = 0.283, *p* < 0.01), warm–cold sensations (*r* = 0.223, *p* < 0.01), and abdominal symptoms (*r* = 0.313, *p* < 0.01), suggesting that slower agility is linked to greater perceived somatic discomfort. Significant positive correlations were further observed between DASS-21 scores and T-Drill agility times (*r* range = 0.307−0.391, *p* < 0.01), indicating that higher emotional distress is associated with slower agility. BlazePod Hits showed weak but significant negative correlations with DASS-21 subscales (e.g., Depression *r* = −0.172, *p* < 0.05), suggesting that better reaction performance is associated with lower emotional distress. Conversely, Reaction Time positively correlated with DASS-21 Total (*r* = 0.203, *p* < 0.01) and GMSCS Total (*r* = 0.118, *p* < 0.01), indicating that slower responses are linked to greater psychological and somatic symptoms.

### 3.2. Analysis of Pretest–Posttest Differences in the SmartACT Group for Motor Variables (T-Drill Agility, BlazePod Hits and Reaction Time)

Paired-sample *t*-tests were used to assess within-group changes in agility (T-Drill agility), BlazePod Hits, and Reaction Time for the SmartACT group. Results are presented in Table 4 and demonstrate significant pre–post improvements across all motor outcomes.

- T-Drill agility improved significantly from pretest (M = 11.80 s) to posttest (M = 10.78 s), MD = −1.07 s, t(68) = 10.29, *p* < 0.001, with a large effect size (d = 2.50, 95% CI [1.79–3.35]).
- BlazePod Hits increased from 16.34 to 19.01, MD = +2.53, t(68) = −5.90, *p* < 0.001, also with a large effect size (d = 1.43, 95% CI [0.87–2.07]).
- BlazePod Reaction Time decreased from 3770.84 ms to 3108.10 ms, MD = −643.75 ms, t(68) = 3.52, *p* < 0.001, with a large effect size (d = 0.85, 95% CI [0.35–1.41]).

These results confirm that SmartACT produced strong and practically meaningful improvements in both agility and visual-motor reactivity.

#### 3.2.1. Motor Agility—T-Drill

T-Drill agility times in the SmartACT group improved significantly from pretest (M = 11.80 s) to posttest (M = 10.78 s), with a mean difference of −1.07 s, t(68) = 10.29, *p* < 0.001. This change corresponds to a large effect size (d = 2.50, 95% CI [1.79–3.35]), confirming the strong impact of the intervention on agility. The MAC and Control groups showed minimal or no change.

#### 3.2.2. Visual Response—BlazePod Hits

BlazePod Hits increased significantly in the SmartACT group from 16.34 to 19.01 (*MD* = +2.53, *t*(68) = −5.90, *p* < 0.001). This improvement reflects a large effect size (*d* = 1.43, 95% CI [0.87–2.07]), indicating substantial gains in visual reactivity. No comparable improvements were observed in the MAC or Control groups.

#### 3.2.3. Reaction Time—BlazePod Reaction Time (RT)

Reaction Time significantly decreased in the SmartACT group from 3770.84 ms to 3108.10 ms (*MD* = −643.75 ms, *t*(68) = 3.52, *p* < 0.001). This corresponds to a large effect size (*d* = 0.85, 95% CI [0.35–1.41]), demonstrating faster psychomotor responses following the intervention. No meaningful changes occurred in the MAC or Control groups.

### 3.3. Repeated Measures ANOVA: Pretest–Posttest Evolution in the Three Groups for Motor Variables and Psychological Indicators

To further investigate the differential effects of the interventions, repeated-measures ANOVAs were conducted for each outcome, followed by Tukey’s post hoc tests where appropriate. The results are presented below for motor variables (T-Drill agility, BlazePod Hits, Reaction Time) and psychological indicators (DASS-21, GMSCS).

#### 3.3.1. Group Comparison Regarding T-Drill Agility Test

A repeated-measures ANOVA was conducted on T-Drill agility scores (*n* = 168) to assess changes in agility across time and between groups, followed by Tukey’s post hoc comparisons. Results showed a significant main effect of time, F(1,165) = 13.14, *p* < 0.001, and a significant time × group interaction, F(2,165) = 20.29, *p* < 0.001, indicating that agility improvements varied across groups. Tukey’s post hoc test (Table 5) revealed that the SmartACT group outperformed both the MAC group (MD = −1.11, *p* < 0.001, η^2^*p* = 0.19, 95% CI [0.09–0.29]) and the Control group (MD = −1.07, *p* < 0.001, η^2^*p* = 0.18, 95% CI [0.08–0.28]). No significant difference emerged between MAC and Control (MD = 0.04, *p* = 0.987, η^2^*p* = 0.00).

**Figure 4:**
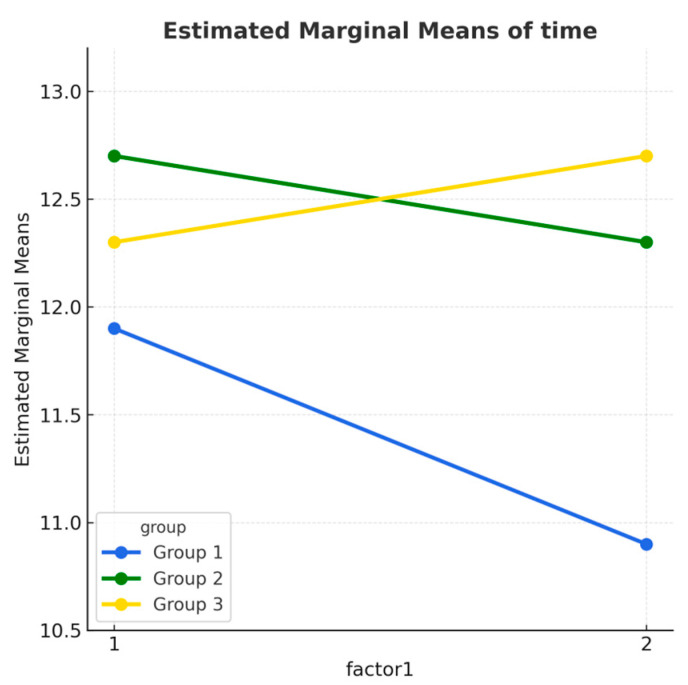
Mean T-Drill agility execution time (seconds) at pretest and posttest across the three groups (SmartACT, MAC, Control). Group 1—SMARTACT—is marked in blue, group 2—MAC—is represented in green, and group 3—control—is indicated in yellow.

The graphical representation of the execution times in the T-Drill Agility Test for the three groups, at the pre- and post-test assessments, is illustrated in Figure 4.

#### 3.3.2. Group Comparison Regarding DASS-21 Total

DASS-21 and GMSCS data were collected from all 193 participants at both pretest and posttest time points. Tukey’s post hoc analysis (Table 6) showed that posttest DASS-21 scores were significantly lower in the SmartACT group compared to both the MAC group (*MD* = −6.43, *p* = 0.002, η^2^*p* = 0.11, 95% CI [0.03–0.21]) and the Control group (*MD* = −4.87, *p* = 0.029, η^2^*p* = 0.07, 95% CI [0.01–0.16]). No significant difference was observed between the MAC and Control groups (*p* = 0.697, η^2^*p* = 0.01).

**Figure 5:**
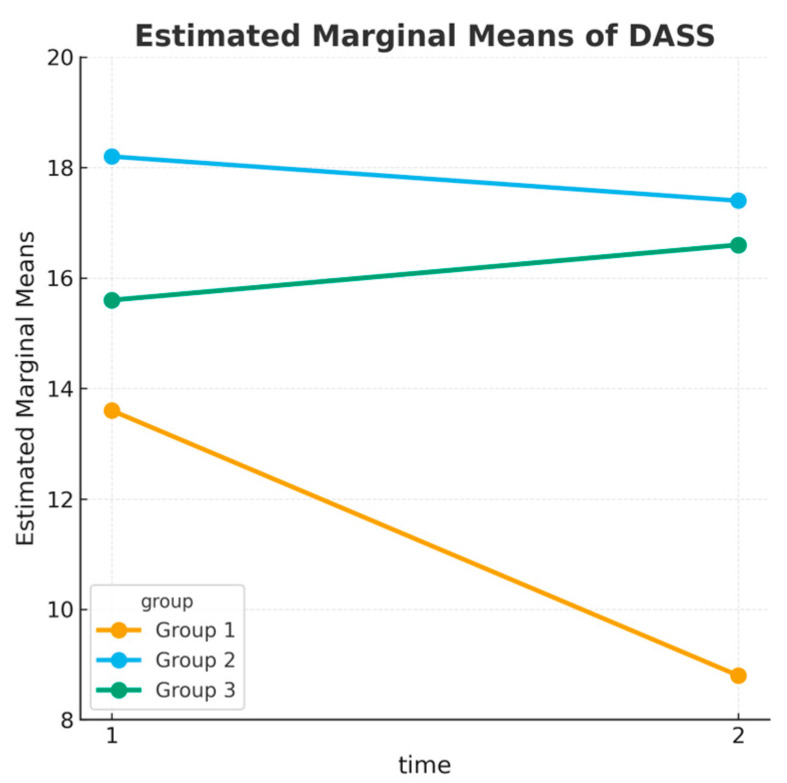
Mean DASS-21 Total scores at pretest and posttest across groups. A larger reduction is evident in the SmartACT group.

These differences confirm that the intervention applied to the SmartACT group had a superior impact compared to the other groups regarding the Depression, Anxiety and Stress Scale (DASS-21), as illustrated in Figure 5. The magnitude of these differences was moderate-to-large (η^2^*p* = 0.07−0.11), indicating not only statistical significance but also practical relevance.

#### 3.3.3. Group Comparison Regarding GMSCS Total

Somatic symptoms (GMSCS Total) decreased significantly in the SmartACT group (from 38.91 to 30.54), with smaller reductions in the MAC group (49.69 to 45.00) and negligible changes in the Control group (42.85 to 40.98). The Tukey post hoc test (Table 7) revealed significant differences between groups. Specifically, SmartACT showed greater efficacy than MAC (MD = −12.62, *p* < 0.001, η^2^*p* = 0.15, 95% CI [0.07–0.25]) in reducing somatic symptoms. The difference between SmartACT and Control was also significant (MD = −7.19, *p* = 0.050, η^2^*p* = 0.08, 95% CI [0.01–0.17]), suggesting a relevant benefit compared to the absence of intervention.

In contrast, no significant differences were identified between the MAC and Control groups (*p* = 0.186, η^2^*p* = 0.03), indicating that the MAC intervention did not substantially impact somatic symptoms in this context.

**Figure 6:**
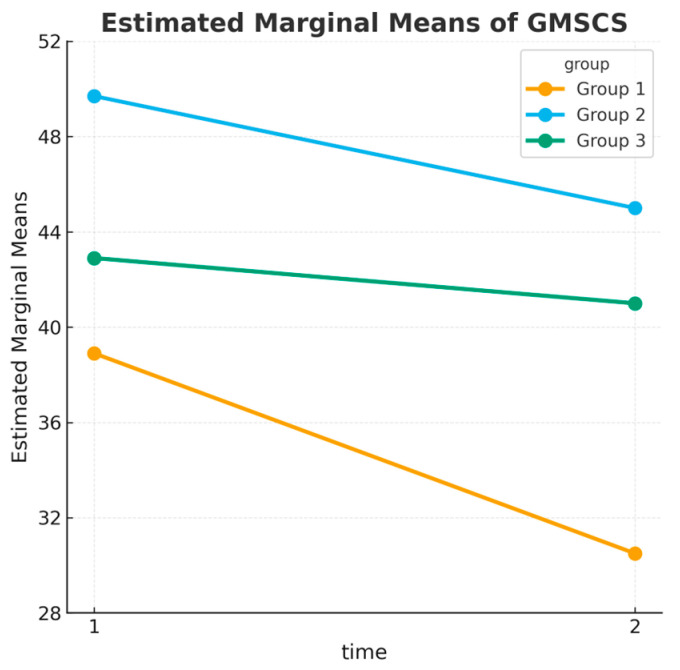
GMSCS Total scores at pretest and posttest across groups. The SmartACT group exhibited the greatest reduction in somatic symptoms.

GMSCS scores decreased significantly over time, suggesting an overall improvement in somatic symptomatology among participants. Group 1 (SmartACT) experienced significantly fewer symptoms compared to Group 2 (MAC) and marginally fewer than Group 3 (Control), as also highlighted in Figure 6. Although the interaction between time and group did not reach statistical significance (*p* = 0.080), post hoc analysis confirmed that Group 1 benefited most from the intervention, with effect sizes in the moderate-to-large range (η^2^*p* = 0.08–0.15). This indicates that SmartACT produced not only statistically significant but also practically relevant reductions in somatic complaints.

#### 3.3.4. Group Comparison Regarding GMSCS Fatigue

Fatigue scores significantly declined over time in all groups (*p* < 0.001), though post hoc analysis revealed that SmartACT led to significantly greater reductions than the other conditions. The mean difference between SmartACT and MAC was −4.88 (*p* < 0.001), and between SmartACT and Control was −2.91 (*p* = 0.014), confirming the superior effectiveness of SmartACT.

No significant differences were observed between MAC and Control (*p* = 0.143). Table 8 presents in detail the results of the Tukey post hoc test for the comparison of perceived fatigue scores (GMSCS) between groups.

#### 3.3.5. Group Comparison Regarding GMSCS Stomach/Abdominal Symptoms

The Tukey post hoc test showed a significant difference between SmartACT and MAC (*MD* = −1.84, *p* = 0.014), indicating fewer stomach/abdominal symptoms in the SmartACT group. The comparison between SmartACT and Control did not reach significance (*p* = 0.272), and no difference was found between MAC and Control (*p* = 0.449) (Table 9).

#### 3.3.6. Group Comparison Regarding GMSCS Head/Shoulder Symptoms

Tukey’s post hoc test (Table 10) showed a statistically significant difference between SmartACT and MAC (*MD* = −2.36, *p* < 0.001, η^2^*p* = 0.13, 95% CI [0.05–0.23]), indicating that participants in the SmartACT group reported fewer head/shoulder symptoms. Differences between SmartACT and Control were not significant (*MD* = −1.02, *p* = 0.194, η^2^*p* = 0.04), nor were differences between MAC and Control (*MD* = 1.34, *p* = 0.067, η^2^*p* = 0.05). These findings confirm that SmartACT was significantly more effective than MAC in reducing head/shoulder pain.

#### 3.3.7. Group Comparison Regarding GMSCS Warm–Cold Symptoms

All three groups experienced decreases in scores between pretest and posttest, suggesting an overall reduction in somatic Warm–Cold symptoms, although the differences between groups were not statistically significant. The most pronounced reduction was observed in the SmartACT group, where the score decreased from approximately 7.7 to 6.0.

This indicates a clear effect of the intervention in this group, contributing to the reduction in perceived thermal discomfort at the body level. The MAC and Control groups experienced smaller decreases of approximately one point.

#### 3.3.8. Group Comparison Regarding GMSCS Heart/Chest Symptoms

Although the SmartACT group showed the greatest reductions in scores on the Heart/Chest subscale, the differences between groups were not statistically significant. This suggests a general tendency toward improvement across all groups, without evidence of a specific intervention effect.

## 4. Discussion

The present findings indicate that psychological well-being and somatic symptomatology are closely linked to motor performance in adolescent athletes. SmartACT produced meaningful gains in agility and visual–motor reactivity—evidenced by shorter T-Drill agility times and better BlazePod performance—together with larger reductions on DASS-21 and GMSCS than both the MAC and Control conditions. This pattern suggests that an integrated, performance-oriented psychological program can enhance athletic readiness by acting simultaneously on attentional control, emotion regulation, and perceived somatic discomfort. Given the magnitude of the pre–post changes observed in the SmartACT group, these effects are likely to be practically relevant for youth athletes and their coaches. These outcomes align with contemporary models that conceptualize agility as primarily reactive, with changes in direction triggered by sport-specific stimuli and constrained by perceptual–cognitive demands [3,4,5,6,7,9,10,11,12,13,15]. Faster reaction to external cues is a hallmark of higher competitive levels in open-skill sports, and measures that capture stimulus–response speed are considered informative for talent identification and training monitoring [4,5,6,7,15,68]. The improvements on BlazePod indices therefore fit within this framework, adding experimental evidence that targeted psychological work can translate into better reactive behavior on the field.

A plausible mechanism for SmartACT’s superiority over MAC is its hybrid structure, which combines ACT processes with guided imagery and hypnosis. Prior work shows that these techniques can strengthen attentional focus, emotion regulation, and self-efficacy, and can facilitate access to optimal performance states such as relaxation and flow [43,44,45,46,49,50,51,58,59,60,61,62,72]. Imagery and hypnosis have also been associated with improvements in technical execution and competitive precision across several sports, including cycling, shooting, golf, football, basketball, and volleyball [50,53,54,55,56,57,73]. In parallel, reductions in GMSCS scores observed here are consistent with evidence that mindfulness/ACT-based methods and imagery can attenuate psychophysiological stress responses and improve interoceptive awareness, thereby lowering the salience of somatic complaints [44,58,59,60,61,62,74,75].

By contrast, the smaller changes in the MAC group may reflect dose and age-fit issues. Seven weeks may be insufficient for adolescents to consolidate mindfulness and acceptance skills, which often require longer daily practice to yield robust transfer to performance contexts [58,72]. In addition, adolescents may engage more readily with techniques that feel directly sport-embedded (imagery, hypnosis, performance routines) than with introspective exercises, leading to stronger motivation and adherence in SmartACT [58,60,61]. These considerations, together with the current data, support the utility of hybrid, performance-linked mental training for youth athletes.

### 4.1. Literature Context and Theoretical Framing

The present study extends literature that ties agility and reaction time to perceptual–cognitive factors and sport-specific stimuli [3,4,5,6,7,9,10,11,12,13,15,68] by showing that a brief, structured psychological intervention can enhance these motor outcomes alongside mental-health indicators. It also integrates strands of evidence on ACT/mindfulness and on imagery/hypnosis in sport: ACT-based approaches reduce emotional symptoms and improve regulation under stress [58,59,60,61,62,72], while imagery and hypnosis improve focus, confidence, and flow, with demonstrated benefits for performance execution [49,50,51,53,54,55,56,57,73]. Taken together, these findings align with models of cognitive–motor integration in which attentional control and affective regulation act as proximal drivers of skilled action under time pressure [44,45,75]. Within this framework, SmartACT likely operates through complementary pathways—top-down regulation (ACT), sensorimotor simulation (imagery), and autonomic/attentional tuning (hypnosis)—to yield concurrent gains in motor efficiency and reductions in psychosomatic distress [44,49,50,51,58,59,60,61,62,74,75,76].

### 4.2. Practical Implications

From an applied perspective, these findings support integrating brief, structured mental training alongside routine physical preparation in youth sport. Coach-delivered micro-sessions that combine ACT-consistent skills (present-moment attention, acceptance, values-guided actions) with sport-specific imagery, concise hypnosis scripts, and competition routines can help athletes translate psychological regulation into faster, more efficient responses in open-skill contexts—i.e., improved T-Drill agility and stimulus–response speed consistent with agility and perceptual–cognitive models [14,15], and with device-based reactivity work [4,68]. Evidence that ACT/mindfulness improves regulation under stress [58,59,60,61,62], and that imagery/hypnosis can enhance focus, flow, self-efficacy, and execution across sports, provides a mechanistic rationale for this hybrid approach in adolescents [49,50,51,54,57,73]. For implementation, sessions can be embedded twice weekly for ~10–15 min during warm-ups or cooldowns over 7 weeks, using simple scripts aligned with technical goals (e.g., cue-based focusing, brief imagery of sport-specific decision points, and short relaxation/hypnosis inductions appropriate for minors and delivered or overseen by trained staff) [49,50,51,54,57,58,59,60,61,62,73]. Programs should be integrated with existing load- and recovery-monitoring to address perceived fatigue and somatic complaints—domains linked to performance and injury risk—using low-burden tools (e.g., DASS-21, GMSCS) alongside standard fatigue practices [40,41,77,78,79,80,81]. Motor tracking can rely on field-friendly tests already used here (T-Drill, BlazePod “Color Catch”) to ensure that psychological skills training remains performance-embedded and ecologically valid [4,15,66,68].

### 4.3. Limitations and Future Directions

Several limitations should be acknowledged. The lack of random assignment limits internal validity, and although baseline equivalence was partly ensured, unmeasured confounders may have influenced the results. Moreover, the absence of a long-term follow-up prevents conclusions about durability, and reliance on self-report for psychological variables introduces potential response bias. Future research should prioritize randomized controlled designs, incorporate objective physiological markers (e.g., HRV, cortisol), and include follow-ups to assess maintenance of change. It will also be valuable to stratify by sport type (team vs. individual; endurance vs. power-based) to account for differing cognitive-motor and emotional demands, and to test dose–response relations to identify the minimal effective dose for adolescent athletes.

## 5. Conclusions

Agility and visual reaction speed are decisive components of sport performance in open-skill disciplines that demand rapid responses to unpredictable stimuli. In this controlled trial, the SmartACT program produced greater gains in T-Drill agility and BlazePod performance than both MAC and Control, together with larger reductions in DASS-21 and GMSCS. For somatic symptoms, all groups improved over time, but the decreases were consistently greatest in SmartACT, indicating added value beyond usual practice and a mindfulness-only approach. While the quasi-experimental design and lack of follow-up limit causal and durability inferences, the magnitude and consistency of effects support the utility of SmartACT as a complement to standard training. Future studies should test SmartACT in randomized designs, include longer follow-ups, and examine differential responses across sport types and competitive levels.

## Figures and Tables

**Table 3:** Correlations between motor, psychological and psychosomatic variables.

	1	2	3	4	5	6	7	8	9	10	11	12
1.T-Drill agility												
2.BlazePod Hits	−0.400 **											
3.BlazePod React. Time	0.371 **	−0.894 **										
4.GMSCS Head Should	0.214 **	−0.075	0.112									
5.GMSCS Heart Chest	0.172 *	−0.049	0.140	0.670 **								
6.GMSCS Stom Abd	0.313 **	−0.106	0.114	0.471 **	0.502 **							
7.GMSCS Warm Cold	0.223 **	−0.030	0.038	0.500 **	0.531 **	0.607 **						
8.GMSCS Fatigue	0.283 **	−0.052	0.085	0.592 **	0.602 **	0.565 **	0.658 **					
9.GMSCS TOTAL	0.295 **	−0.074	0.118	0.780 **	0.810 **	0.764 **	0.806 **	0.879 **				
10.DASS-21 Depression	0.391 **	−0.172 *	0.189 *	0.492 **	0.529 **	0.450 **	0.431 **	0.623 **	0.635 **			
11.DASS-21 Anxiety	0.307 **	−0.147 *	0.151 *	0.574 **	0.674 **	0.452 **	0.568 **	0.650 **	0.726 **	0.715 **		
12.DASS-21 Stress	0.368 **	−0.189 *	0.213 **	0.548 **	0.549 **	0.444 **	0.519 **	0.677 **	0.688 **	0.766 **	0.767 **	
13.DASS-21 TOTAL	0.386 **	−0.186 *	0.203 **	0.588 **	0.637 **	0.491 **	0.552 **	0.712 **	0.747 **	0.909 **	0.902 **	0.927 **

**. Correlation is significant at the 0.01 level (2-tailed); *. Correlation is significant at the 0.05 level (2-tailed).

**Table 4:** Significant pretest–posttest differences in the SmartACT group for the analyzed motor variables (T-Drill agility, BlazePod Hits, BlazePod Reaction Time).

Variable	Time	M	MD	*T* (df)	*p*	Cohen’s *d* [95% CI]
T-Drill agility (s)	Pretest	11.80	−1.07	10.29 (68)	<0.001	2.50 [1.79, 3.35]
	Posttest	10.78				
BlazePod Hits	Pretest	16.34	+2.53	−5.90 (68)	<0.001	1.43 [0.87, 2.07]
	Posttest	19.01				
BlazePod Reaction Time (ms)	Pretest	3770.84	−643.75	3.52 (68)	<0.001	0.85 [0.35, 1.41]
	Posttest	3108.10				

Note. MD = mean difference; *t* = t statistic; *p* = probability value; *d* = Cohen’s d; CI = confidence interval.

**Table 5:** Results of the Tukey post hoc test for comparing T-Drill Agility scores (sec) between groups.

Compare Groups	MD	SE	*p*	η^2^*p* (95% CI)
SmartACT vs. MAC	−1.11	0.226	0.000	0.19 [0.09–0.29]
SmartACT vs. Control	−1.07	0.232	0.000	0.18 [0.08–0.28]
MAC vs. Control	0.04	0.246	0.987	0.00 [0.00–0.04]

Note. MD = mean difference; SE = standard error; *p* = probability value; η^2^p = partial eta squared; CI = confidence interval.

**Table 6:** Results of the Tukey post hoc test for comparing DASS-21 Total between groups.

Compare Groups	MD	SE	*p*	η^2^*p* (95% CI)
SmartACT vs. MAC	−6.43	1.844	0.002	0.11 [0.03–0.21]
SmartACT vs. Control	−4.87	1.891	0.029	0.07 [0.01–0.16]
MAC vs. Control	1.56	1.918	0.697	0.01 [0.00–0.06]

Note. MD = mean difference; SE = standard error; *p* = probability value; η^2^p = partial eta squared; CI = confidence interval.

**Table 7:** Results of the Tukey post hoc test for comparing GMSCS Total scores between groups.

Compare Groups	MD	SE	*p*	η^2^*p* (95% CI)
SmartACT vs. MAC	−12.62	2.968	0.000	0.15 [0.07–0.25]
SmartACT vs. Control	−7.19	3.045	0.050	0.08 [0.01–0.17]
MAC vs. Control	5.43	3.088	0.186	0.03 [0.00–0.09]

Note. MD = mean difference; SE = standard error; *p* = probability value; η^2^p = partial eta squared; CI = confidence interval.

**Table 8:** Results of the Tukey post hoc test for comparing GMSCS fatigue scores between groups.

Compare Groups	MD	SE	*p*	η^2^*p* (95% CI)
SmartACT vs. MAC	−4.88	.998	0.000	0.17 [0.08–0.27]
SmartACT vs. Control	−2.91	1.024	0.014	0.09 [0.01–0.18]
MAC vs. Control	1.97	1.038	0.143	0.04 [0.00–0.11]

Note. MD = mean difference; SE = standard error; *p* = probability value; η^2^p = partial eta squared; CI = confidence interval.

**Table 9:** Results of the Tukey post hoc test for comparing GMSCS Stomach/Abdominal scores between groups.

Compare Groups	MD	SE	*p*	η^2^*p* (95% CI)
SmartACT vs. MAC	−1.84	0.647	0.014	0.08 [0.01–0.17]
SmartACT vs. Control	−1.03	0.664	0.272	0.03 [0.00–0.09]
MAC vs. Control	0.81	0.673	0.449	0.02 [0.00–0.07]

Note. MD = mean difference; SE = standard error; *p* = probability value; η^2^p = partial eta squared; CI = confidence interval.

**Table 10:** Results of the Tukey post hoc test for comparing GMSCS Head/Shoulder scores between groups.

Compare Groups	MD	SE	*p*	η^2^*p* (95% CI)
SmartACT vs. MAC	−2.36	0.574	0.000	0.13 [0.05–0.23]
SmartACT vs. Control	−1.02	0.588	0.194	0.04 [0.00–0.11]
MAC vs. Control	1.34	0.597	0.067	0.05 [0.00–0.12]

Note. MD = mean difference; SE = standard error; *p* = probability value; η^2^p = partial eta squared; CI = confidence interval.

## Data Availability

The data presented in this study are available on request from the corresponding author due to privacy and ethical restrictions.

## Abbreviations

The following abbreviations are used in this manuscript:

MAC	Mindfulness Acceptance Commitment
SmartACT	Smart Acceptance and Commitment Therapies
DASS-21	Depression, Anxiety and Stress Scales (21-item)
GMSCS	Ghent Multidimensional Somatic Complaints Scale
RT	Reaction Time
CI	Confidence Interval
ANOVA	Analysis of Variance
η^2^p	partial eta squared
MD	Mean Difference
SD	Standard Deviation
